# Cost of Utilising Maternal Health Services in Low- and Middle-Income Countries: A Systematic Review

**DOI:** 10.34172/ijhpm.2020.104

**Published:** 2020-06-28

**Authors:** Aduragbemi Banke-Thomas, Francis Ifeanyi Ayomoh, Ibukun-Oluwa Omolade Abejirinde, Oluwasola Banke-Thomas, Ejemai Amaize Eboreime, Charles Anawo Ameh

**Affiliations:** ^1^Department of Health Policy, London School of Economics and Political Science, London, UK.; ^2^Centre for Global Child Health, The Hospital for Sick Children (SickKids), Toronto, ON, Canada.; ^3^Health Education West Midlands, Birmingham, UK.; ^4^Department of Medicine, University of Alberta, Edmonton, AB, Canada.; ^5^Centre for Maternal and Newborn Health, Liverpool School of Tropical Medicine, Liverpool, UK.

**Keywords:** Maternal Health, Utilisation, Cost, Economic Evaluation, Developing Countries

## Abstract

**Background:** Cost is a major barrier to maternal health service utilisation for many women in low- and middle-income countries (LMICs). However, comparable evidence of the available cost data in these countries is limited. We conducted a systematic review and comparative analysis of costs of utilising maternal health services in these settings.

**Methods:** We searched peer-reviewed and grey literature databases for articles reporting cost of utilising maternal health services in LMICs published post-2000. All retrieved records were screened and articles meeting the inclusion criteria selected. Quality assessment was performed using the relevant cost-specific criteria of the Consolidated Health Economic Evaluation Reporting Standards (CHEERS) checklist. To guarantee comparability, disaggregated costs data were inflated to 2019 US dollar equivalents. Total adjusted costs and cost drivers associated with utilising each service were systematically compared. Where heterogeneity in methods or non-disaggregated costs was observed, narrative synthesis was used to summarise findings.

**Results:** Thirty-six studies met our inclusion criteria. Many of the studies costed multiple services. However, the most frequently costed services were utilisation of normal vaginal delivery (22 studies), caesarean delivery (13), and antenatal care (ANC) (10). The least costed services were post-natal care (PNC) and post-abortion care (PAC) (5 each). Studies used varied methods for data collection and analysis and their quality ranged from low to high with most assessed as average or high. Generally, across all included studies, cost of utilisation progressively increased from ANC and PNC to delivery and PAC, and from public to private providers. Medicines and diagnostics were main cost drivers for ANC and PNC while cost drivers were variable for delivery. Women experienced financial burden of utilising maternal health services and also had to pay some unofficial costs to access care, even where formal exemptions existed.

**Conclusion:** Consensus regarding approach for costing maternal health services will help to improve their relevance for supporting policy-making towards achieving universal health coverage. If indeed the post-2015 mission of the global community is to "leave no one behind," then we need to ensure that women and their families are not facing unnecessary and unaffordable costs that could potentially tip them into poverty.

## Introduction

Despite significant progress made during the 15-year span of the Millennium Development Goals, the burden of maternal morbidity and mortality remains highest in low- and middle -income countries.^1^ By the commencement of the Sustainable Development Goals (SDGs) era in 2015, sub-Saharan Africa (66%) and Southern Asia (20%) accounted for over 80% of global maternal deaths (254000), with about 243 women per 100000 live births dying from mostly preventable pregnancy-related causes.^1^ The consensus target for the next decade is to reduce maternal mortality ratio to 70 per 100000 live birth and newborn mortality ratio to below 12 per 1000 live births.^2^ However, multiple factors including weak health systems, socio-economic disparities and poor planning and monitoring data partly explain the lag in progress.^3^

As has been well established, maternal health services required to meet the new SDG target include antenatal care (ANC), skilled birth attendance for normal vaginal delivery and emergency obstetric care (EmOC), post-natal care (PNC), family planning and post-abortion care (PAC) (Described in detail in Supplementary file 1).^4^ However, high poverty levels in many low and middle-income countries worsens health disparities such that even when health services are available, they are inaccessible due to high costs. Analysis shows that between 1990 and 2015, the poorest women accounted for the highest proportion of maternal deaths, increasing from 68% to 80%.^5^ Indeed, poor financing mechanisms for health service utilisation have led to an increase in out-of-pocket payments and catastrophic health expenditure, resulting in families shouldering the costs of maternal healthcare beyond their capabilities, often with trans-generational ramifications. This further widens health and socio-economic disparities, while creating additional barriers to health service utilisation.^6^

In an attempt to bridge financial barriers to maternal service quality and utilisation, health-financing schemes such as no-fee-for-service policies, pay-for-performance and voucher packages have been implemented over the years,^7^ however, maternal health coverage remains sub-optimal. Under the mandate of universal healthcare coverage, the goal is to ensure that all people can access quality healthcare without being exposed to catastrophic health expenditure.^8^ To comprehend the full complexity of financial factors that influence maternal health service utilisation in low- and middle-income countries (LMICs), and its effect on individual users and their families, it is necessary to identify and disaggregate the cost implications of maternal health service utilisation, as this cost is most reflective of the burden experienced by service users. Such information will be useful in understanding the financial barriers to access and highlight areas where maternal healthcare can be re-structured to prevent prohibitive costs to users. Furthermore, it would be useful for reforming health systems and service modalities to meet the necessary targets for universal health coverage. The objective of our review, therefore, is to systematically assess and summarise the available evidence on costs of utilising maternal health services in LMICs.

## Materials and methods

###  Study Design

A systematic review was conducted following the Preferred Reporting Items for Systematic Reviews and Meta-Analyses (PRISMA) approach.^9^ The checklist depicting how this review aligns with the PRISMA approach is presented in Supplementary file 2. Guidance for conducting systematic reviews on costs and cost-effectiveness of interventions from the Centre for Reviews and Dissemination,^10^ the Task Force on Community Preventive Services,^11^ and the Joanna-Brigg’s Institute^12^ were applied.

###  Search Strategy

We searched multiple databases including the African Journal Online, CINAHL Plus, EconLit, Embase, Global Health Archive, Google Scholar, LILACS, Popline (until September 1, 2019, when the website was retired), ProQuest, PubMed, Scopus and Web of Science for articles published from January 2000 to September 2019, as the costs data within this period were deemed to be more current and relevant for planning services in the SDG era. In searching, both medical subject headings (MeSH) and/or key words were combined, using Boolean operators “OR” within categories and “AND” between the three categories of words/phrases that captured the interventions of interest (maternal health services (‘ante*natal care’ OR ANC OR ‘birth’ OR ‘skilled birth attendance’ OR ‘obstetric emergenc*’ OR ‘emergency obstetric care’ OR EmOC OR ‘caesarean*’ OR ‘vacuum’ OR ‘post*natal care’ OR ‘PNC’ OR obstetric OR delivery OR maternity OR ‘family planning’ OR contraception)), their costs (‘cost*’ OR ‘cost of care’ OR ‘cost*analysis’ OR ‘cost*effectiveness’ OR ‘cost*utility’ OR ‘cost*benefit’ OR ‘economic evaluation’) and the setting of interest (all LMICs). Search terms were chosen and combined using an approach that guaranteed an optimal strategy for retrieving cost and economic studies of maternal health services.^13^

 We also searched websites of governments, non-government organisations, United Nations agencies, and institutions that were likely to have reported costing of maternal health services including Averting Maternal Death and Disability, FP2020, Guttmacher Institute, LMIC Ministries of Health, Management Sciences for Health, Maternal Health Task Force, Population Council, United Nations Children’s Fund, United Nations Fund for Population and the World Health Organization (WHO).

 In addition to the automated search, we also searched for other potentially relevant articles by reviewing the reference lists of retrieved articles. If a study was found in the grey literature, which was later published in the peer-reviewed literature, the peer-reviewed version was selected for the purposes of our review. We limited our search to studies published in English and French languages, both of which are languages familiar to the review team. The search was conducted independently by 3 authors (ABT, FA, and OBT), with search results compared for completeness. We conducted the search between June 30, 2019 and September 30, 2019,

###  Selection of Studies

 After duplicates were identified and removed, 2 co-authors (ABT and FIA) independently screened titles and abstracts (or executive summaries for grey literature) of the retrieved records for relevance and eligibility, based on the set inclusion/exclusion criteria (defined below). If titles or abstracts/summaries were deemed relevant, full text were reviewed to verify relevance of study for the review. Full texts were subsequently stored in shared folders within an automated reference manger, Mendeley Desktop version 1.19.4 (Elsevier, Amsterdam, The Netherlands). Justification for inclusion or exclusion of studies was documented in a pre-developed Microsoft Excel (Microsoft Corporation, Redmond, USA) worksheet. Any discrepancies regarding the relevance of studies for the review were resolved through discussions with the senior author (CAA), who is a subject matter expert.

###  Inclusion and Exclusion Criteria

Economic evaluation studies typically report cost data,^14^ which is the interest of our review. For this review, we included articles that:

- were economic evaluation studies of any or a combination of the maternal health services along the continuum of care from the perspective of women or their households.
- presented data on cost of any of the maternal health services collected from one or multiple LMICs, as defined by the World Bank as countries with gross national income per capita <US$12375 in 2018,^15^ whether or not the cost data that they provided were presented as a lump sum or disaggregated into cost components such as consultation fees, laboratory tests, drugs/medications etc.

However, we excluded articles that:

- presented lump costs that could not be disaggregated into service-specific costs (ANC, skilled birth attendance, EmOC, PNC, PAC, and family planning).
- were commentaries, editorials, letters and costing studies based on models and projections of utilisation costs as well as those that did not specifically mention the service being costed. For example, those that stated that they costed service use of “maternal complications” without stating the specific complication.
- reported costs of services which are part of the continuum of care but focused on newborn, children or adolescents.^4^
- focused on cost of implementing policies to improve maternal health service utilisation and those for which it was difficult to separate cost to utilisation of services to mothers from those specifically for their newborns.
- took a mixed (patient and provider) perspective and for which it was not possible to disaggregate cost associated with either perspective.
- presented cost categories only (for example, $0-$500) as opposed to actual costs, as well as studies published after year 2000 using cost data preceding year 2000.

###  Quality Assessment of Included Studies

The 24-item Consolidated Health Economic Evaluation Reporting Standards (CHEERS) checklist has been extensively used for assessing quality of reporting full economic evaluations.^16^ However, as this review included mostly partial economic evaluation studies which capture purely cost data,^14^ only the cost-focused criteria in the CHEERS checklist were applied. This choice is based on quality assessments used in similar cost-focused reviews.^17^ For each item, a score of 1 was awarded if the criterion is fully met, 0.5 if partially met, 0 if not met or if only minimal information was provided, and NA if not applicable. The total score achieved across all the criteria was subsequently summed-up and converted to percentages. Following the classification used in similar published reviews,^17,18^ studies which met 75% or more of the criteria fully were classified as high quality, 50%-74% as average quality and below 50% as poor quality. Each included study was independently assessed by 2 co-authors (ABT and FIA).

###  Data Extraction

Guidance on approach and content for data extraction were sought from a previous review and an expert opinion.^19,20^ We extracted relevant data into a pre-developed Microsoft Excel (Microsoft Corporation, Redmond, USA) sheet. For all included articles, we collected data on the article description (authors, year of publication, article title, journal, and stated objective), study setting (country of study and scale of study [‘facility level’ – one facility in one district, ‘district level’ – multiple facilities in one district, ‘sub-national level’ – multiple facilities across many districts, ‘national level’ – multiple facilities across an entire country; ‘multi-national level’ – multiple facilities across multiple countries]), site of study (household vs. facility), country of organisation conducting study, study participants, study design (partial vs. full economic evaluation; standalone vs. nested study), costing of maternal health services (intervention(s) costed, facility type (health centre, hospital), facility ownership (private, public or mission), number of facilities, component of cost included (for example, cost of labour, equipment, medicines, supplies and where applicable, opportunity cost),^14^ year of costs data, currency, stated exchange rate used for analysis if cost was converted to US$) as well as findings reported (including mean or median cost of service utilisation estimates).

In extracting data, we made some key considerations. If cost data was collected across 2 years (for example, 2002-2003), without specific information on cost of which year was used in analysis, we selected the more recent year. If more than two years were studied, we took the mid-year. Also, when costs analysis for a specific service was sub-grouped, (for example by district in a national survey), we selected summary measures that reflected costs of the entire sample. For papers that presented cost at different facility types as well as an overall summary costs that reflected costs across all facility types, we selected the disaggregated costs by each facility type, as this was more relevant for our analysis. Where costs were stated in both local currency and US$ equivalents, we selected local costs, as recommended by costing experts.^21^ For studies that reported US$ equivalents of costs of service utilisation but did not state the exchange rate used, we applied for the average annual exchange rate for the year of costing.^22^

 When specific data was missing from retrieved articles, we made attempts to contact the study authors directly via contact information provided in the study, or by using portals such as ResearchGate and LinkedIn. Data extraction was conducted independently by 2 co-authors (ABT and FIA) and then checked for accuracy by 2 other co-authors (IOA and OBT).

###  Data Analysis and Synthesis

 Characteristics of included studies were summarised, and their presented cost data authors were collated. Summary findings were presented using tables and charts (ABT, OBT and EAE).

Using a subgroup analysis, we sought for the different cost components associated with the singular use of specific maternal health service. To do this, firstly, we identified studies that disaggregated total service cost into cost components, as these were more valid and valuable for the purposes of our review. We were also particularly interested in studies that specified the disaggregated cost by facility type (for example, dispensary, health centre, clinic and hospital). Secondly, leveraging guidance on adjustments for inflation and currency changes for health economic studies,^23^ we converted the local currency value of all component costs of service utilisation to US$ equivalents using official OANDA Corporation exchange rates.^22^ We then inflated these costs using the gross domestic product implicit price deflator for the year of costing as stated in the study and our base year, 2019.^24^ All cost were presented in US$, as opposed to international dollars (I$), as the US$ currency is widely understood and is the medium of exchange for many international transactions.^23^ Based on these adjusted US$ equivalents, total cost of utilisation per service estimates were calculated by summing up the component adjusted costs. Subsequently, we compared our newly derived adjusted total costs across studies and tried to explain any observed patterns, taking cognizance of any methodological differences and where possible, differences in the details of the care package received by women, as reported in the included studies. In doing this, we also highlighted the major cost drivers for service utilisation.

 Included articles that presented lump costs which could not be disaggregated into cost components were analysed separately. For such studies, we calculated the newly adjusted utilisation cost per service estimates using the same approach as with disaggregated cost. Although these adjusted costs were not included in our analysis since they could not be disaggregated, they were used for the narrative synthesis conducted as part of this review.

In line with global guidance for conducting systematic review of economic evaluations,^12^ by reviewing individual studies in detail and implementing targeted searches of the peer-reviewed and grey literature, we explored population contextual and intervention design characteristics that could help explain our findings.

## Results

**Figure 1:**
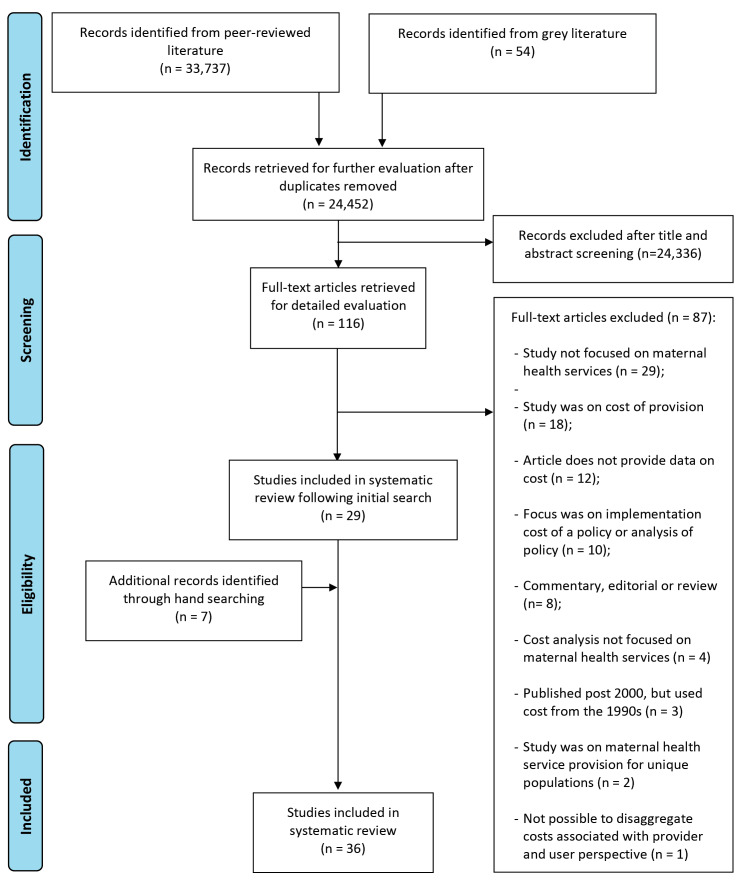
PRISMA diagram. Abbreviation: PRISMA, Preferred Reporting Items for Systematic Reviews and Meta-Analyses

A total of 24452 articles from peer-reviewed and grey literature sources were screened by title and abstract for inclusion in the full-text review. Following removal of duplicates, full text of 116 articles were read, of which 30 articles met the inclusion criteria. Six additional articles were identified by hand-searching the bibliography of the included articles, bringing to a total of 36 studies, all retrieved from peer-reviewed sources, and included in the final review.^25-60^ No study was found in the grey literature that met our inclusion criteria. The PRISMA diagram showing the flow chart of our search findings is presented in Figure 1.

###  Overview of Studies

**Figure 2:**
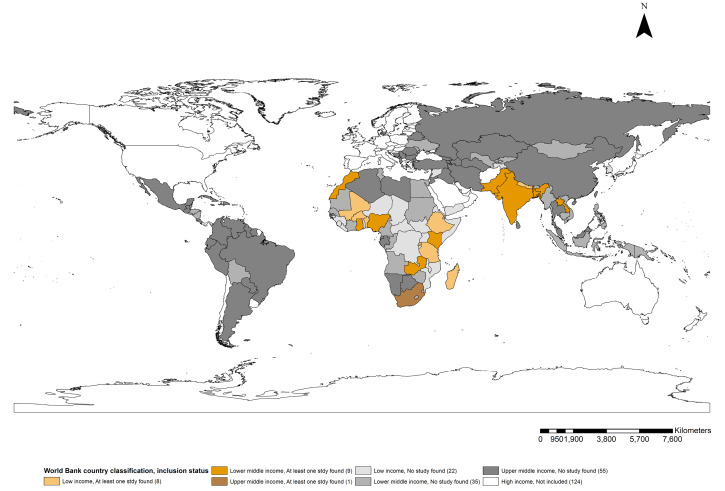
Geographical Distribution of LMICs With at Least 1 Published Cost of Maternal Health Service Utilisation Study. Abbreviation: LMICs, low- and middle-income countries.

Ten studies were published between 2000 and 2010^25,27,28,38,39,41,48,51-53^ while the remaining 26 were published post-2010.^26,29-37,40,42-47,49,50,54-60^Figure 2 shows the geographical distribution of LMICs having at least one published study on the cost of maternal health service utilisation. Fifteen low-income countries have published costing studies focused on maternal health service utilisation. This includes three each in Nepal^42,46,53^ and Tanzania,^37,41,52^ two each in Ethiopia^32,55^ and Mali,^40,57^ and one each in Benin,^51^ Burkina Faso,^38^ Madagascar,^43^ Pakistan,^36^ and Rwanda.^50^ Twenty lower-middle income countries have published costing studies including nine studies published on India,^25,28-30,33,34,47,48,56^ four studies in Nigeria,^27,45,49,59^ two each in Bangladesh,^31,39^ and Zambia,^58,60^ and one each in Ghana,^51^ Kenya,^41^ Lao PDR,^44^ and Morocco.^54^ One study was conducted in an upper-middle-income country, South Africa.^26^

Included studies reported cost of single or multiple maternal health services. In all, twenty-two studies reported costs of utilising skilled birth attendance for normal vaginal delivery^25,28-30,32-34,36,38,39,41,42,44-49,51-53,56^ and another two assessed cost associated with using assisted vaginal delivery.^32,59^ Fourteen studies estimated cost of utilising skilled birth attendance during caesarean delivery^25,31,32,36,39,42-44,48,49,53,54,56^ while ten studies focused on cost of ANC utilisation.^25,28,30,31,35,37,47,49,51,59^ Five studies focused on cost of utilising PNC^25,28,30,31,49^ with another five costing the use of safe abortion or PAC.^27,32,55,58,60^ Two studies costed utilisation of at least one of the non-delivery-related EmOC signal functions.^26,40,51^ Details of the services for which utilisation costs have been reported in each study are presented in the data extraction sheet attached as Supplementary file 3.

With respect to the scale of the studies, eleven of the 36 studies were done at a sub-national scale,^25,27,30,31,41,47,49,50,52,53,57^ four studies were conducted on a national scale,^28,29,32,34^ while eight studies involved multiple facilities within one district.^35,37-40,51,54,59^ Thirteen studies were conducted within a single facility.^26,33,36,42-46,48,55,56,58,60^

###  Quality Assessment of Included Studies

Quality assessment of included studies is detailed in Supplementary file 4. Seventeen studies were assessed as high quality.^25,26,30,34,36,37,40,42,44,46,47,50–52,54,58,60^ Fourteen studies were assessed to be of average quality.^29,31–33,38,39,41,43,45,49,53,55,57,59^ Five studies were judged to be of low quality.^27,28,35,48,56^ For the lower scoring articles, the criteria warranting the least scores were those related to detailing a breakdown of costs incurred for utilising the intervention and incorporation of indirect costs in their analysis.

###  Methods Used in Collecting and Analysing Cost Data in Included Studies

Table provides an overview of the methods used in the costing studies included in this review. Twenty-five studies used a cross-sectional survey.^25,27,31-33,36,40-54,56,57,59,60^ Four studies used ethnographic methods,^35,37-39^ three used secondary data analysis,^28,29,34^ two used a qualitative study design with interviews only^30,58^ and one each used cost-effectiveness analysis^26^ and the PAC costing methodology^55^ respectively. In nine studies, cost data collection was nested within a larger study.^32,35,37,38,40,50,51,57,60^ For the remaining 27 studies, data was collected primarily for the purposes of the costing study.^25-31,33,34,36,39,41-49,52-56,58,59^ Most of the studies collected data from the women or their relatives or used data from surveys that engaged women directly.^25-31,33-54,56-60^ However, a few studies asked health workers to report on the cost of service utilisation.^32,55^

**Table:** Overview of Methodology Used in Included Costing Studies

**Study Characteristics**	**No. of Studies**	**% Of Total**
Study design (n = 36)		
Cross-sectional study with surveys	25	69.4
Ethnography	4	11.1
Secondary data analysis	3	8.3
Phemenological qualitative study design with interviews	2	5.6
PAC costing methodology	1	2.8
Cost-effectiveness analysis	1	2.8
Type of study (n = 36)		
Stand-alone	27	75.0
Nested	9	25.0
Source of data collection (n = 36)		
Women	34	94.4
Health workers	2	5.6
Currency of presentation (n = 36)		
Local currency only	20	55.6
US$ only/local currency and US$ equivalent	16	44.4
Disaggregation of service cost (n = 36)		
Disaggregated/possible to disaggregate by service	22	61.1
Not disaggregated/not possible to disaggregate by service	14	38.9
Opportunity cost of service utilisation included (n = 22)		
Included	7	31.8
Not included	15	68.2
Summary measure of cost or provision (n = 36)		
Mean	33	91.7
Mean and median	3	8.3

Abbreviation: PAC, post-abortion care.

Twenty studies presented their cost analyses in the local currency of the study country,^25,27,31,33,36,38,39,42,43,45,46,48,49,52,53,56-60^ while the remaining 16 studies used US dollar equivalents.^26,28-30,32,34,35,37,40,41,44,47,50,51,54,55^ Twenty-two of the 36 studies provided cost breakdown that could be disaggregated by the specific service provided.^26,27,31,34,36,37,39-48,51,52,54,55,59,60^ The remaining 14 studies either did not provide disaggregated cost or it was not possible to disaggregate cost based on the available data.^25,28-30,32,33,35,38,50,53,56-58^ Of the 22 studies with disaggregated costs, seven included opportunity costs of service utilisation.^26,36,44,46,48,51,54,60^ Three studies presented median cost in addition to the mean (see Supplementary file 3).^30,33,51^

###  Cost of Utilising Maternal Health Services

####  Cost of Utilising Ante-natal and Post-natal Care

Following inflation to 2019 US dollars, reported total cost of utilising ANC in the literature (including disaggregated and non-disaggregated costs) ranged from US$0.01 in a public clinic in Rwanda^50^ to US$78.28 in a private hospital in India.^47^ Estimated median total cost of utilising ANC in hospitals in India and Bangladesh is US$14.78, while in a clinic or health centre in India, Nigeria, Rwanda, and Tanzania, estimated median ANC utilisation cost was US$2.41.

**Figure 3:**
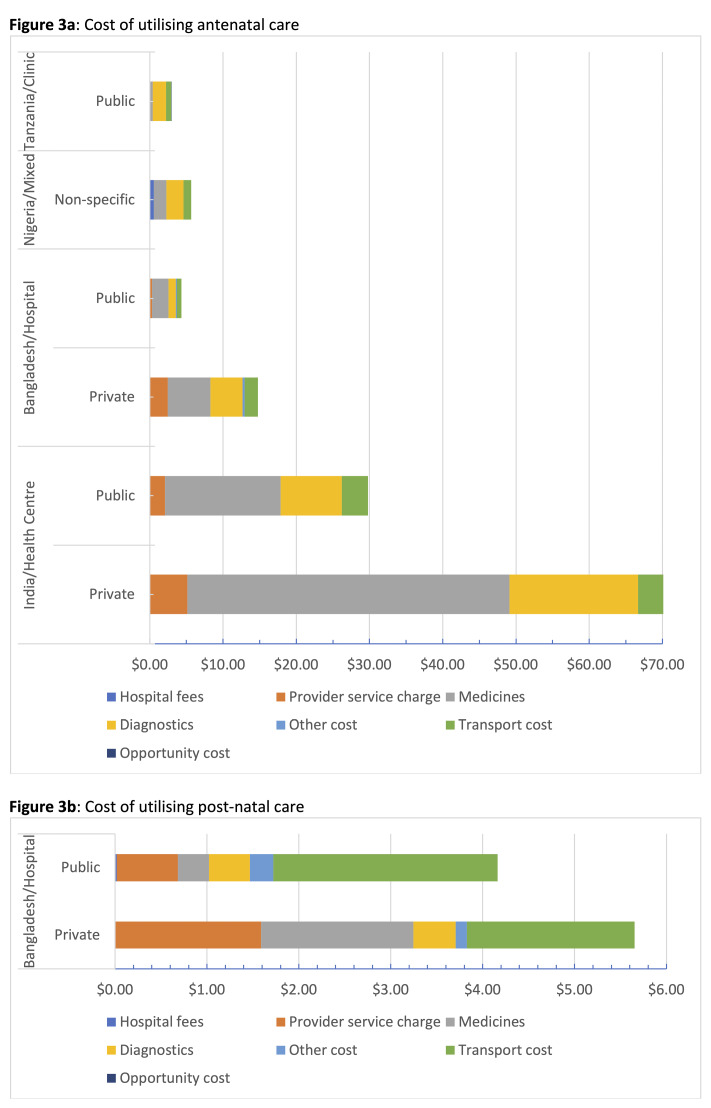
Disaggregated Costs of Utilising (a) ANC and (b) PNC. Abbreviations: ANC, antenatal care; PNC, post-natal care.
 Note: For Tanzania, the included study only estimated and reported cost for public sector utilisation. For Nigeria, the term “Non-specific” was used as the included study did not differentiate public from private facilities.

Adjusted disaggregated costs of utilising ANC and PNC are presented in Figure 3 and Supplementary file 3. Summing these adjusted disaggregated cost, which included facility-based fees, transport and opportunity costs, total facility-based service utilisation cost ranged from US$2.21 at a public clinic in Tanzania^37^ to US$66.68 in a private hospital in India.^47^ Medicines and diagnostics were the main cost drivers for ANC. Women who had to pay some form of ANC registration fees paid between US$0.02 in Bangladesh^31^ and US$0.57 in Nigeria.^59^ The studies in both India and Tanzania did not report any registration fees.^37,47^ Cost of transportation to and from the facilities ranged from US$0.74 in Tanzania to US$6.60 in India.

There was only one study that presented disaggregated cost of utilising PNC.^31^ In this study, transport was reported as the major cost driver. For the four studies that presented cost of PNC utilisation as a lump sum,^25,28,30,49^ estimates ranged from US$0.01 when care is received at home^25^ to US$17.62 at a private hospital in India.^28^

####  Cost of Utilising Skilled Birth Attendance During Intra- partum Care

Following inflation to 2019 US dollars, median cost of having a skilled health personnel to support a normal vaginal delivery was US$6.13, US$6.85 and US$8.82 in a mission-owned health centre, nursing home and a private health centre respectively, across the relevant studies. Estimated median cost of having an skill birth attendant (SBA) supported vaginal delivery in Bangladesh, Burkina Faso, Ethiopia, Ghana, India, Lao PDR, Nepal, Nigeria, Pakistan and Tanzania was US$39.94 in a public hospital and US$82.96 in a private hospital. For caesarean delivery, median cost was estimated at US$178.17 in public hospitals and US$188.74 in private hospitals across Bangladesh, Burkina Faso, Ethiopia, Ghana, India, Lao PDR, Madagascar, Mali, Nepal, and Nigeria. Cost of utilising assisted vaginal delivery was only reported in Ethiopia where it ranged from US$1.91 in a public health centre to US$74.23 in a private hospital.^32^

**Figure 4:**
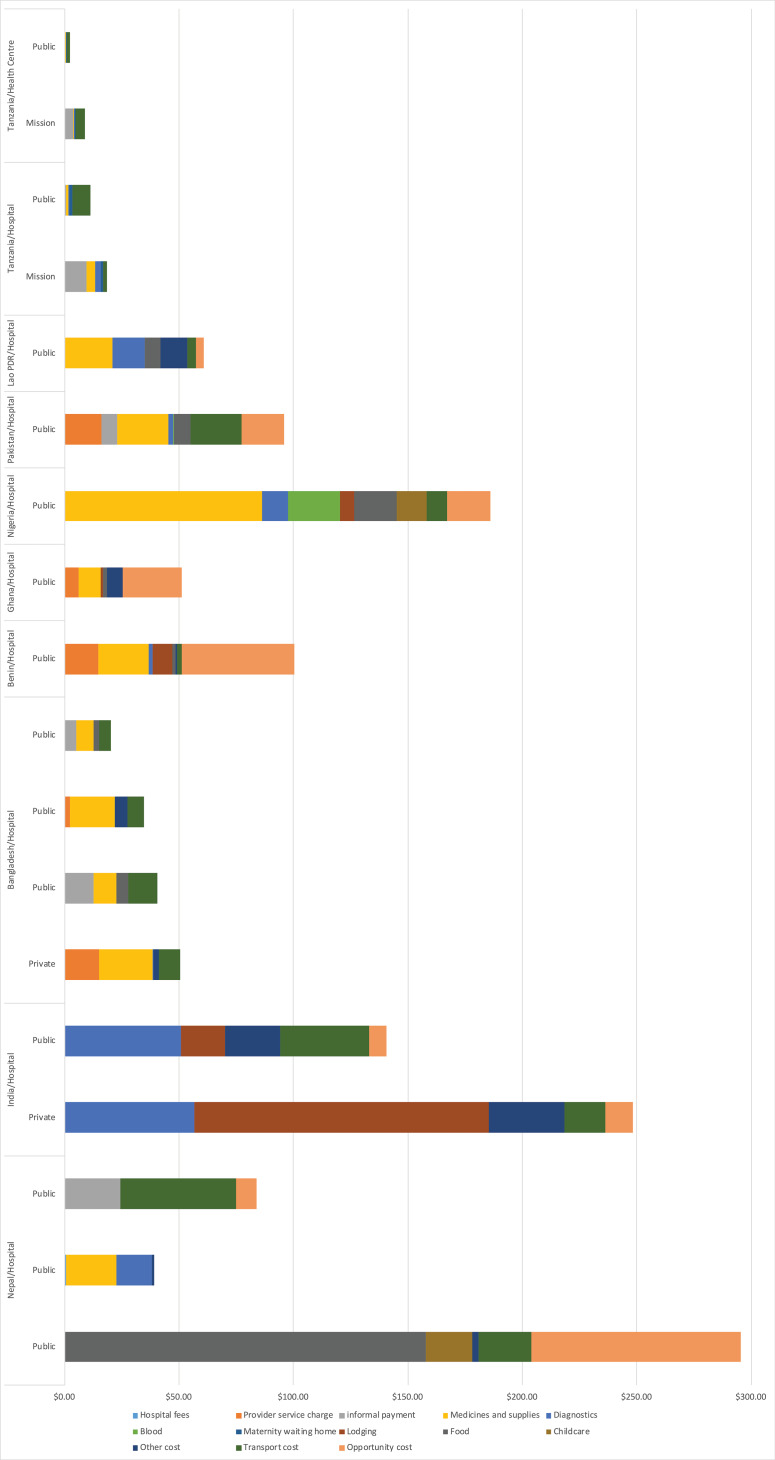
Disaggregated Costs of Utilising Skilled Birth Attendance for Normal Vaginal Delivery.

Adjusted and disaggregated costs of utilising skilled birth attendance for normal vaginal delivery are presented in Figure 4 and Supplementary file 3. For the studies with disaggregated cost for vaginal deliveries, total financial cost of utilising a SBA for normal vaginal delivery ranged from US$0.94 in a public health centre in Tanzania^52^ to US$218.32 in a private hospital in India.^48^ When transportation and opportunity costs are included to reflect the full economic cost of utilisation, cost of utilising a SBA for normal vaginal delivery ranged from US$2.50 in a public health centre in Tanzania^52^ to US$295.34 in a private hospital in Nepal.^46^ Cost drivers varied in different countries, with some reporting one of medicines and supplies, transport, or lodging as the principal cost driver. Provider service charge for normal vaginal delivery which women had to pay to access care in public hospitals ranged from US$2.46 in Bangladesh^31^ to US$16.01 in Pakistan. Informal payments ranging from US$0.30 to US$24.38 were estimated from studies conducted in Bangladesh, Nepal, Pakistan, and Tanzania.^36,39,52,53^

There was only one study that presented disaggregated cost of utilising PNC.^31^ In this study, transport was reported as the major cost driver. For the four studies that presented cost of PNC utilisation as a lump sum,^25,28,30,49^ estimates ranged from US$0.01 when care is received at home^25^ to US$17.62 at a private hospital in India.^28^

####  Cost of Utilising Skilled Birth Attendance During Intra- partum Care

Following inflation to 2019 US dollars, median cost of having a skilled health personnel to support a normal vaginal delivery was US$6.13, US$6.85 and US$8.82 in a mission-owned health centre, nursing home and a private health centre respectively, across the relevant studies. Estimated median cost of having an SBA-supported vaginal delivery in Bangladesh, Burkina Faso, Ethiopia, Ghana, India, Lao PDR, Nepal, Nigeria, Pakistan and Tanzania was US$39.94 in a public hospital and US$82.96 in a private hospital. For caesarean delivery, median cost was estimated at US$178.17 in public hospitals and US$188.74 in private hospitals across Bangladesh, Burkina Faso, Ethiopia, Ghana, India, Lao PDR, Madagascar, Mali, Nepal, and Nigeria. Cost of utilising assisted vaginal delivery was only reported in Ethiopia where it ranged from US$1.91 in a public health centre to US$74.23 in a private hospital.^32^

Adjusted and disaggregated costs of utilising skilled birth attendance for normal vaginal delivery are presented in Figure 4 and Supplementary file 3. For the studies with disaggregated cost for vaginal deliveries, total financial cost of utilising a SBA for normal vaginal delivery ranged from US$0.94 in a public health centre in Tanzania^52^ to US$218.32 in a private hospital in India.^48^ When transportation and opportunity costs are included to reflect the full economic cost of utilisation, cost of utilising a SBA for normal vaginal delivery ranged from US$2.50 in a public health centre in Tanzania^52^ to US$295.34 in a private hospital in Nepal.^46^ Cost drivers varied in different countries, with some reporting one of medicines and supplies, transport, or lodging as the principal cost driver. Provider service charge for normal vaginal delivery which women had to pay to access care in public hospitals ranged from US$2.46 in Bangladesh^31^ to US$16.01 in Pakistan. Informal payments ranging from US$0.30 to US$24.38 were estimated from studies conducted in Bangladesh, Nepal, Pakistan, and Tanzania.^36,39,52,53^

**Figure 5:**
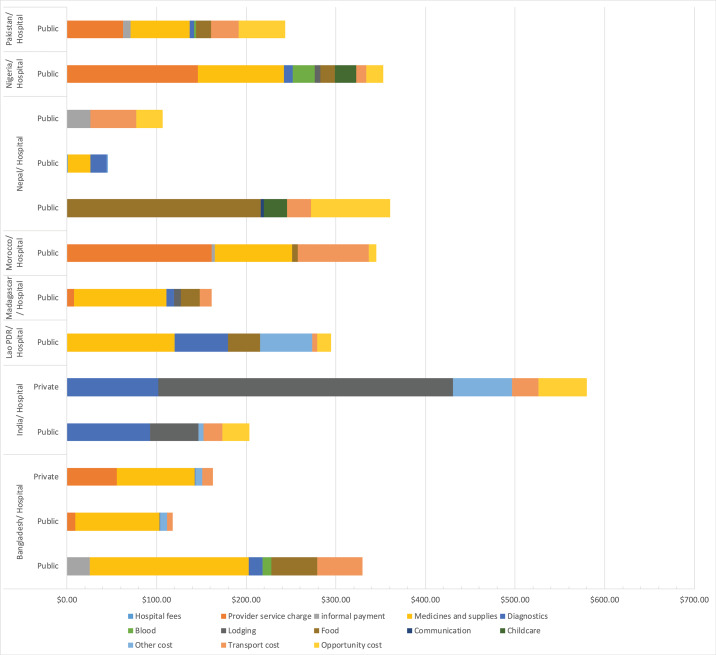
Disaggregated Costs of Utilising Skilled Birth Attendance for Caesarean Delivery.

Adjusted and disaggregated costs of utilising skilled birth attendance for caesarean delivery are presented in Figure 5. For these studies, financial cost of utilisation ranged from US$45.80 in a public hospital in Nepal^42^ to US$496.45 in private hospital in India.^48^ When transportation and opportunity costs are included to reflect the full economic cost of utilisation, cost of utilising a SBA for caesarean delivery ranged from US$106.98 in a public health centre in Nepal^53^ to US$580.19 in a private hospital in India.^48^ Cost drivers varied by country of study. Provider service charge for caesarean delivery where women had to pay to access care in public hospitals ranged from US$8.02 in Madagascar^43^ to US$146.27 in Nigeria.^45^ Informal payments ranging from US$8.89 to US$26.45 were reported in Bangladesh, India, and Nepal (Figure 5 and Supplementary file 3).^36,39,53^

For both vaginal and caesarean deliveries, there were opportunity cost of service utilisation reported, with adjusted estimates ranging from US$3.51 in Lao PDR for normal vaginal delivery^44^ and US$8.81 in Morocco^54^ for caesarean delivery to US$88.78 for caesarean delivery in Nepal.^42^ Of the studies that reported transport cost for vaginal delivery, this ranged from US$0.09 in Tanzania^52^ to US$50.74 in Bangladesh (Figure 5).^39^

####  Cost of Utilising Skilled Birth Attendance for Emergencies and Post-abortion Care

Only one study reported cost of receiving care from an SBA in emergency situations in Benin and Ghana.^51^ Following cost adjustments, in Benin, financial cost of receiving care for dystocia was highest (US$370.37) and care of haemorrhage was US$159.28 while in Ghana, cost of hypertension was highest (US$194.11) and dystocia was least (US$130.79).^51^ Including transport and opportunity costs reveals a total cost of utilisation ranging from US$256.41 for receiving care for dystocia in Ghana to US$754.98 for receiving same care in Benin.^51^ For studies that reported financial cost of utilising PAC, adjusted estimates ranged from US$5.99 in Ethiopia^55^ to US$112.70 in Nigeria.^27^ The study in Nigeria,^27^ which was the upper limit of the financial cost estimates did not include opportunity costs. However, for the two studies that did, opportunity costs of US$13.45 to US$24.13 were reported.^26,60^ Following adjustments, total un-disaggregated cost of care for unsafe abortion was US$86.70 in Zambia while safe abortion in Ethiopia ranged from US$3.81 in a public health centre to US$41.58 in a non-governmental organisation-managed hospital (see Supplementary file 3).^32^

###  Qualitative Themes Emerging From Narrative Synthesis

 Three themes emerged from our synthesis of the available literature. Firstly, several women in LMICs experience significant financial burden of utilising maternal health services. Secondly, there are “other” costs incurred for utilising services in these settings. Finally, “free” care which is being implemented in many LMICs may not actually free. We discuss these themes in detail in the ensuing paragraphs.

####  Financial Burden of Utilising Maternal Health Services in Low- and Middle-Income Countries

In Nigeria, Sambo et al estimated the average cost of ANC and delivery at a public facility (US$22), translating to 2% of the average annual income of household heads in the community.^59^ Cost of a spontaneous vaginal delivery at a teaching hospital in Benin was estimated at 3.4% of annual household expenditure.^51^ Even higher, in Nepal, median patient’s expenditure on hospital based vaginal delivery was 13% of the annual family income.^46^ However, the total cost of obstetric emergencies represented a larger financial burden for households. For, example, in Benin, where the annual household cash expenditure in year 2000 was US$781.6, cases of dystocia posed the greatest financial burden on families, accounting for 23 and 34% of annual household cash expenditure in a public non-teaching and teaching hospital, respectively.^51^ The cost of accessing EmOC in Mali represented up to a quarter of the annual income of the poorest families.^40^ For caesarean delivery, Honda et al reported that out-of-pocket costs as a proportion of annual non-food household expenditure ranged between 32.9% for the higher socio-economic group, 105.3% for the medium socio-economic group and 109.1% for the lower socio-economic group in Madagascar.^43^ Satapathy et al concluded that cost of caesarean delivery is “ *far beyond the limits of an average middle class family in India*”^48^ and in Nigeria, Adamu et al found that the mean expenditure for delivery (US$246.30 = N39400) was more than the monthly family income for 94.6% of respondents included in their study.^45^ Kalu-Umeh et al reported a significant association between the monthly income of women and difficulty in payment for services.^49^

In some cases, payment had to be made before service is received, even in cases of emergencies. In Ethiopia, for example, one-fifth of all facilities with delivery services required payment in advance for an obstetric emergency, including 75% of non-governmental organisation and 30% of public hospitals.^32^ Many women reported difficulties in paying for maternal health services resulting in them either proceeding without treatment, selling an asset, or taking a loan. In Burkina Faso and Tanzania, about a third of women had to sell assets or crops to be able to pay for delivery. Conversely, in Kenya, 79% of women reported that the funds required to pay for their delivery came from their immediate family.^41^ About half of the women that delayed care seeking because of cash considerations experienced avoidable complications including miscarriages.^49^

To cope with the financial burden of paying for delivery, some women had to cut down on their regular spending, more so those from the poorest households.^52^ On average, Kruk et al reported that 40.6% of women cut down on spending.^52^ In Ghana, where first time users had to pay GH¢15.00 (US$2.75) for an ultrasound or scan test, GH¢3.00 (US$0.55) for laboratory tests, and GH¢5.00 (US$0.91) for issuance of a new registration photo identity card, pregnant women who could not afford these payments either returned home, used nonformal providers such as traditional birth attendants or sought care from religious outlets.^35^

####  The “Other” Costs Incurred for Utilisation

Six studies reported that “other” costs are being required of women before they can access maternal health services in LMICs.^30,32,35,39,44,46,51^ In Benin, for example, women are requested to pay to use toilet facilities in the hospital during their hospitalisation.^51^ Though not mandated as per national or hospital policy, in Ghana, after payment has been collected for registration cards, women are then required to purchase items such as baby vest, baby cover cloth, baby diapers, baby lotion, pegs and shower caps, many of which are sold by midwives-on-duty.^35,51^ Also, a teaching hospital in Ghana mandates payment for meals, even if the women have not requested this service.^51^ In Nepal, items such as buckets, mat, mug, soap, thermos flask, toothpaste, and toilet papers are required of women before admission in the hospital for delivery.^46^ In Lao PDR, women had to pay gratuities to health workers before being discharged while women in India had to pay as much as 17% of total costs incurred as indirect expenditure.^30,44^

An ethnographic study in Bangladesh revealed consequences for women and their families, when they do not pay. The author reported that, after assisting a doctor with a delivery, two nurse maids (known locally as ayahs) demanded a 400 Bangladeshi Taka (US$7.25) tip. When the family refused to pay, “ *four or five ayahs crowded round and started to quarrel with the family. Suddenly, the nurses were not able to locate the patient’s file, which the ayahs had hidden.”* This the author noted to be a common occurrence for which, patients and their families were also sometimes deliberately misinformed about the location of ancillary medical services by the nursemaids.^39^

####  “Free” Care Which May not Actually Free

Some studies reported that in countries where policies guaranteed “free” maternity services, many women still had to pay for services out-of-pocket. For example, in Tanzania, despite the free delivery services policy, 62.5% of women still had to pay for delivery services in public facilities (73.0% at government dispensaries, 26.2% at government health centres, and 78.9% at government hospitals).^52^ Specifically, 84.6%, 35.7% and 30% of women who delivered in a government dispensary, government health centres and government hospitals respectively had to pay some provider-levied charges or consultation fees.^52^

In Ghana, where a free maternity care policy was being implemented in the early 2000s, some women reported that they had to pay some money to access ANC. However, women confirmed that direct medical care associated with delivery was actually free in public clinics and hospitals, provided a woman was registered under the National Health Insurance Service scheme.^35^ Similarly, in Morocco, where caesarean delivery was free, it was reported that some public hospitals complied with the policy and offered caesarean delivery for free but this was not the case in teaching hospitals.^54^

In Ethiopia, despite a policy to deliver free maternal health services to women at point-of-use, about 67% of the 751 facilities surveyed nationally had charged women a fee, with a large proportion of these being government health centres. These included US$0.80 for gloves, syringes, and needles and US$1.80 for intravenous fluids and catheters. Women were also charged an average payment of US$0.80, US$2.00, and US$1.10 for life-saving obstetric medicines including oxytocin, penicillin, and gentamicin respectively.^32^

## Discussion

The objective of this review was to systematically assess and summarise the available evidence on costs of utilising maternal health services in LMICs. In the final analysis, we found 36 studies that conducted costing of maternal health service utilisation in LMICs from year 2000 till date with majority of these (n = 30) deemed as average to high quality. Twenty-five of the included studies were published after 2010. The reason for this increased interest is not particularly clear but it may be linked to the increased interest in and implementation of financing schemes aimed at guaranteeing that women do not face catastrophic health expenditure, as part of efforts to achieve universal health coverage in the late-2000s.^7^

As evident from our findings, diverse methods have been used in collecting and analysing utilisation cost for maternal health services in LMICs. For these various methods, their impact on final cost estimates are well recognised.^21^ However, the real issue is not the diversity of methods being used, but the need for more application of best practices for costing so as to improve validity and comparability of results. While most of the studies (94%) in our review collected cost data from women who used the service, others (6%) collected data from health workers.^32,55^ Certainly, enquiring cost of service utilisation from women who actually used the services themselves seems a logical source of data collection, but, as reported by studies in our review, this in itself may be subject to recall bias.^33,51-53,56–58^ On the contrary, asking health workers, who themselves have not paid for using the services means their estimates may not be reflective of the actual cost of utilisation, especially as they may not be aware of or refuse to declare “hidden costs” that women are compelled to pay. As such, best practices like collecting cost data from multiple sources and triangulating such data,^21^ need to be promoted. Other methodological issues, such as presenting both financial (direct cost of utilisation) and economic (indirect and opportunity cost) data, which provides a broader representation of the actual cost of utilising services, as well as the use of median cost as a summary measure of utilisation cost, which, as opposed to the mean is more robust than the outlier costs, need to also be promoted.^21,23,61^ In addition to these considerations, there is the need to improve transparency on what is actually being costed. How many days are women spending in the hospital? Who is providing the care (nurse, doctor or mix of both, which is the most likely scenario)? These are key indicators of service delivery data that have implications on utilisation cost.^62^ If available, these sorts of data can significantly improve comparability of costing studies. In our review, only three studies provided some information on the number of days that the woman spent in hospital for intra-partum-care.^42,46,53^ No study in our review provided information on the cadre of the care provider.

Following cost adjustments, our findings showed that irrespective of the study country, services such as skilled birth attendance and PAC were more expensive to women and their families and constituted a higher percentage of household income than preventive services such as ANC and PNC. For example, we found that the median cost of utilising skilled birth attendance for normal vaginal delivery is four times more expensive than ANC in the public health centres. In private hospitals, median cost of caesarean delivery was two and a half times more than normal vaginal delivery. With EmOC, median cost ranged from three to ten times of cost incurred for normal deliveries. Women who survived complications in pregnancy reported as much as three times higher cost of using skilled health personnel than did women with uncomplicated deliveries, even in the same hospitals.^38^ Even though some have suggested that the cost of preventive services are deliberately set low to encourage utilisation,^63^ the variation in cost is still significantly higher for delivery and EmOC services to warrant specific attention. This higher cost implication to access more complex and usually more critical care packages in the continuum of maternal health services probably explains some of the difficulty that women experience in accessing delivery services provided skilled health personnel, especially amongst the poor, as has been shown in other studies.^64,65^

In terms of cost of service utilisation as it relates to facility type, we found that, for the most part, there was an increment in utilisation cost of maternal health service from public to private facilities for ANC, skilled birth attendance and PNC. For example, women who delivered in a mission facility paid nearly four times more on direct medical costs than women who delivered in a public facility.^52^ This is probably explained by the fact that mission facilities are also classed as part of the private sector,^66^ which require funds to sustain service provision. In addition, we also observed an increment in utilisation cost of service utilisation from lower-level facilities such as health centres and clinics to higher level facilities like hospitals. This finding is not particularly unexpected. Indeed, there is a case to encourage women to attend care at these lower level public facilities, especially as some women have reported preference for the higher-level facilities, because of their higher confidence in the skill of providers working in such facilities.^67^ For country-specific cost variations for public v. private sectors, see Supplementary file 3. If the consideration given by more than 56% of women in Nepal to want to save money on delivery and transport cost by delivering at home is anything to go by,^53^ then there is a need to design policy responses that are tailored to encourage women to choose lower-level facilities. Services at this level of care need to be affordable and the facilities themselves must be sufficiently equipped, stocked with supplies and staffed by SBAs, thereby ensuring that quality is not compromised.

Upon disaggregation of cost of service utilisation, it appears the major cost drivers varied by provider and by setting. For example, in Tanzania, where there was a free maternity service policy in place, the cost of delivery in public facilities was principally driven by transport costs and unofficial fees charged of the women by health providers. However, in the mission facilities, the major drivers were the direct cost of service provision paid by women including provider fees, medicines, laboratory tests and supplies.^52^ In another study, conducted in Pakistan, the two major cost components for spontaneous vaginal delivery were transportation and drugs, each contributing 23% to total cost of utilisation, whereas medicines (27%) and hospital fees (26%) were the largest cost components of caesarean delivery.^36^ While varied, it is important that individual health systems identify major cost drivers of maternal health service utilisation for women and respond with appropriate cost saving policies such as transportation and/or care vouchers, solicitation of private sector contributions to support cost of care of the most vulnerable women and user fee exemptions.

However, we found that in the context of most user fee exemption policies in LMICs, “free” care may not actually be free. In Ethiopia, Ghana, and Tanzania,^32,35,52^ women still had to pay some formal and/or informal fees to access delivery care. Some of the payments being made in the context of “free” service have been associated with misinformation created by the government through the media.^35^ Certainly, the government needs to clearly spell out information about “free” maternal health services to ensure that the populace get the right message and the policy achieves its projected objectives.^68^ In addition, clarity is needed for governments themselves on the rationale for implementing user fee removal policy, as such clarity also helps frame messaging to the populace. There is some evidence to suggest that removal of user fees leads to increase in facility delivery,^69^ however, there is limited evidence that this increase leads to improvement in health outcomes.^70,71^ Indeed, some authors have suggested that removal of user fees can result in supply gaps in medicines and supplies for women, overworked and demoralised care providers and poorer overall quality of care.^72^ While efforts to reduce financial burden should be sustained to ensure that the poorest and the most vulnerable women continue to seek care, it is important that countries implementing free services are clear about what is free and set up monitoring systems to ensure that women are not paying out of pocket for care that is otherwise designated as free. Lack of clarity only deepens distrust of and disinterest in engaging with formal care.^73,74^

In addition, the “other”/unofficial/informal/under-the-counter costs that are being charged of women also need to be removed completely and where they are being charged illegally, there is a need to set up legal protections for women. Tips to staff were reported in Lao PDR and in India, where such payments constituted as much as 17% of total indirect expenditure.^30,44^ Of course, there will be those who willingly want to pay these informal costs, such as in Morocco, where women made such payments “ *because they wanted the staff to share in their joy on the occasion of the birth, or to help non-medical staff*.”^54^ However, it is the forceful demand for informal payments with consequences on care that needs to be addressed. Other costs such as use of toilet facilities, and sundry items for mother and baby during delivery,^32,35,39,46,51^ are payments that can be excluded from the financial burden that women have to manage to access maternal health services in LMICs. Parkhurst and Ssengooba identified these informal payments as a potential barrier to the utilisation of maternal health services, especially when such services have been deemed to be free.^73^ This is contrary to the objective of fee exemption policies. To reduce demand for these informal payments, evidence from a Cochrane review point to interventions such as internal control practices at facility level and increased transparency and accountability for co-payments combined with reduced incentives as being potentially effective.^75^

###  Strengths and Limitations

To the best of our knowledge, this is the first systematic review which focuses on the cost of utilising maternal health services in LMICs. We searched for costing studies published in both peer-reviewed and grey literature, inflating all cost to comparable 2019 US dollar equivalents of cost data from multiple countries and disaggregating cost components for the various services. Doing this, allowed us to for the first time make some meaningful comparisons with regards to costs of service utilisation across the continuum of care. However, there were some limitations. Despite our best efforts, it was not possible to accurately ascertain that we were comparing like-for-like in all settings, as we could not fully describe the specific package of care provided to women in all included studies, especially as it relates to the care provider and the number of days spent receiving the care. However, by setting the inclusion criteria to only select studies published from year 2000, we ensured that we were comparing like-for-like services in terms of design, as global guidance regarding care packages were updated on or around this period. For example, ANC became packaged as focused ANC.^76^ Finally, it is important to bear in mind that the findings from our review can only be as good as the results of and information available from the included studies. To address this, we reached out to authors in cases in which we had some missing information.

## Conclusions

While there is a case for more consensus building around methodology to be used for costing, we have shown in this review that with appropriate adjustments, it is possible to make some sensible comparisons between costing studies, especially for more preventive services such as ANC and PNC. For maternal health service utilisation, costing studies can serve as a good starting point for curating and learning from existing approaches and gleaning lessons to improve woman-centred quality care irrespective of socio-economic status of women in LMICs. If indeed the mission of the SDG era is to “ *leave no one behind*,” then we need to ensure that women are not facing sometimes unjustifiable, unnecessary or unaffordable costs to utilise maternal health services in LMICs.

## Ethical issues

 Not applicable.

## Competing interests

 Authors declare that they have no competing interests.

## Authors’ contributions

 ABT, IOA, OBT, and CAA conceptualised the review. ABT and FIA conducted literature searches, screened them for inclusion and conducted the quality assessment. Discrepancies were resolved through discussions with CAA. ABT, IOA, and AF reviewed the included studies and were involved in data extraction. Data synthesis was conducted by ABT, OBT, and EAE. ABT, IOA, EAE, and CAA were involved in interpretation of data. All authors were involved in drafting the manuscript and approved the final version.

## Authors’ affiliations

^1^Department of Health Policy, London School of Economics and Political Science, London, UK. ^2^Centre for Global Child Health, The Hospital for Sick Children (SickKids), Toronto, ON, Canada. ^3^Health Education West Midlands, Birmingham, UK. ^4^Department of Medicine, University of Alberta, Edmonton, AB, Canada. ^5^Centre for Maternal and Newborn Health, Liverpool School of Tropical Medicine, Liverpool, UK.

## Supplementary files


Supplementary file 1. Description of Maternal Health Services and Care Packages.

Supplementary file 2. PRISMA Checklist.

Supplementary file 3. Data Extraction Sheet.

Supplementary file 4. Quality Assessment of Included Studies.
